# Supplementary material for: tpHusion: An efficient tool for clonal pH determination in Drosophila
Source: PLoS One. 2020 Feb 14;15(2):e0228995. doi: 10.1371/journal.pone.0228995 (PMC7021318; doi:10.1371/journal.pone.0228995)
Supplement: Attachment — Submitted filename: Response to reviewers.pdf [file pone.0228995.s001.pdf]

Response to reviewers

We would like to thank both reviewers for raising critical points and for constructive feedback, which helped us to improve our manuscript. In particular, we have introduced the following changes:

Reviewer #1: This manuscript by Gupta and Stocker examines the usefulness and efficiency of a new genetic tool that assesses the intracellular pH of cells. The study describes a practical follow-up to pH detectors that are already in use. The major difference of the tool generated in this work is that it functions independent of the Gal4/UAS binary gene expression system. The authors demonstrate the use of tpHusion in live, fixed, and clonal tissues.

Overall, this study is relevant for three reasons. (1) The tpHusion tool can detect intracellular pH in live and fixed tissues independent of Gal4/UAS. (2) tpHusion can be used along with binary expression systems to evaluate intracellular pH in mosaic tissue. (3) tpHusion can be used to assess intracellular pH across different tissues, developmental stages, and biological processes. However, there are a few issues that should be addressed:

(1) *The figures and figure legends can be better annotated to highlight different regions of the tissue. Dashed lines, arrowheads, or brackets can be used to indicate various regions within the tissue. For example, on the eye discs, a dashed line along the morphogenetic furrow can more clearly show the amf versus the pmf. Similarly, arrowheads pointing out the FSCs versus the follicle cells allows for better interpretation of the distribution of the tpHusion signal in the ovarioles. The wing disc and the larval brain images can also benefit from outlining and labeling the different compartments.*

The suggested annotations have been introduced in Figures 2 and 3. Arrows and arrowheads indicate follicle cells and FSCs, a dashed line labels the morphogenetic furrow, the pouch and notum have been clearly separated, and brackets have been used to denote the different regions of the larval brain. Corresponding information has also been included in the associated figure legends.

(2) *The dashed squares in the figures are difficult to see. Replace them with solid squares of a different color or represent another way. It would be helpful to have the squares on the images in the single channels too.*

The color of the dashed square has been replaced with yellow, and the squares are also added to the single channel images.

(3) *Explain the calibration bar a bit more – specifically the colors and the associated numbers.*

The calibration bar represents the relative ratio of SEpHluorin and FusionRed intensities within a tissue from low (blue) to high (red). The limits are set to clearly denote the differences over the entire tissue area.
The above information is now included in the Materials and Methods section.

(4) *Increase the magnification of the ovarioles in figure 2. The magnification of the ovarioles differs between figure 2 and figure 3. At the higher magnification used in figure 3, it is easier to see the cells and the tpHusion signal.*

The magnification of ovarioles in Figure 2 has been increased (similar to Figure 3). The corresponding changes to the scale are indicated in the figure legend.

(5) *Include low magnification images of the single channels of figure 4 for an unbiased view of the distribution of the tpHusion signal across clones throughout the entire wing disc.*

We excluded the low magnification images because the membrane localization and distribution of tpHusion is difficult to view at lower magnification. For reference, a panel of discs containing *LacZ*, Ras^{V12}-overexpressing and *Tsc1*-knockdown clones is shown in Figure R1.

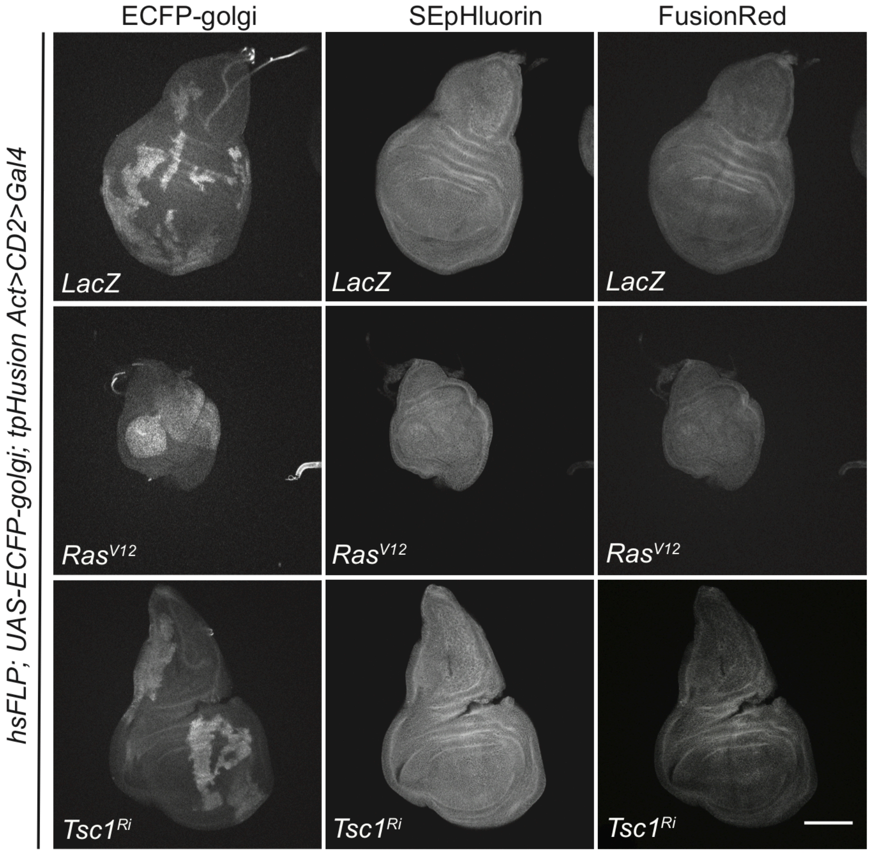

Figure R1. Clonal variation of tpHusion in wing imaginal discs. SEpHluorin and FusionRed signal in wing discs containing (A) *LacZ*-overexpressing, (B) Ras^{V12}-overexpressing and (C) *Tsc1* knockdown clones. Clones are labeled by Golgi-specific ECFP. Scale bar = 100 µm.

If the reviewer prefers, we can include a similar figure for the different conditions tested in Figure 4 as a supplementary figure.

(6) *The authors state that the detection of intracellular pH in fixed tissue is similar to that observed in live tissue. However, there appears to be a significant reduction of tpHusion*

signal in the images of the fixed eye discs. Based on the images presented, the tpHusion signal in the fixed larval brain appears expanded and more intense. Finally, the tpHusion signal in fixed ovarioles seems shifted with more intense FusionRed signal compared to SEpHluorin signal in fixed compared to live tissues. Discuss these differences.

The comparison has been made between the SEpHluorin to FusionRed intensities ratio of different regions. The absolute pHi values cannot be determined for the fixed tissues, so a direct comparison with live tissues is not possible. However, the variability in pHi of different regions within a tissue is conserved based on ratio of intensities. The corresponding statement has also been made clearer in the text.

Additional minor points
(1) Change HCO3 to HCO3-.
(2) Page 6, Line 122: Insert (NDS) after Normal Donkey Serum.

The requested changes have been made in the manuscript.

Reviewer #2: Gupta et al. describe the development of tpHusion, a membrane associated cytosolic pH sensor. The authors describe their strategy, which relies on expressing the sensor under the control of the tubulin promoter in order to obviate the necessity of the GAL4/UAS system. The utility of the strategy was validated by measurements of pH in different tissues during development, and in both fixed as well as live tissues.

The manuscript is technically sound, and the authors' conclusions are backed by their data. This tool will certainly be useful to researchers interested in studying cytosolic changes during development. There remains, however, one minor concern that could be addressed by additional experiments as detailed below. tpHusion is tagged to the membrane using an HRAS-sequence. Though this is a reasonable strategy to attach the sensor to the PM, there is a concern regarding localization of the sensor to specific domains at the PM. Attachment of HRAS to the membrane is dependent upon the levels of membrane cholesterol (i.e. lipid rafts). Under conditions of alterations in PM cholesterol levels, the sensor could fall off the membrane. How would this change the pH measurements? It is recommended that the authors describe the effects of removing PM cholesterol with beta-cyclodextrin on the recordings made with tpHusion. The goal of this endeavor would be to provide a framework that future researchers could use should they worry about expressing the sensor in backgrounds that exhibit alterations in cholesterol trafficking.

To address the reviewer's concerns about variations in pHi with cholesterol levels in the plasma membrane, we tested the ratio of SEpHluorin and FusionRed intensities after incubation of live wing discs with methyl-β-cyclodextrin (MβCD), as suggested. Starting with 10 mM MβCD concentration (Dason and Charlton, 2014), we did not observe any change in the SEpHluorin and FusionRed intensities or their ratio (Figure R2) up to 50 min after MβCD incubation. Upon incubation with higher concentrations, no change was observed with 25 mM MβCD, whereas a significant disruption of tissue architecture was observed at 50 mM MβCD after 30 min. Due to the lack of a positive control to test for loss of cholesterol levels

at lower concentrations of MβCD, we decided not to include the data in the manuscript. However, we do agree that the tpHusion reporter has to be used with caution to compare pHi in cells with different cholesterol levels. Further work will be required to establish the behavior of tpHusion in dependence of membrane cholesterol levels but we feel that this is beyond the scope of our present manuscript.

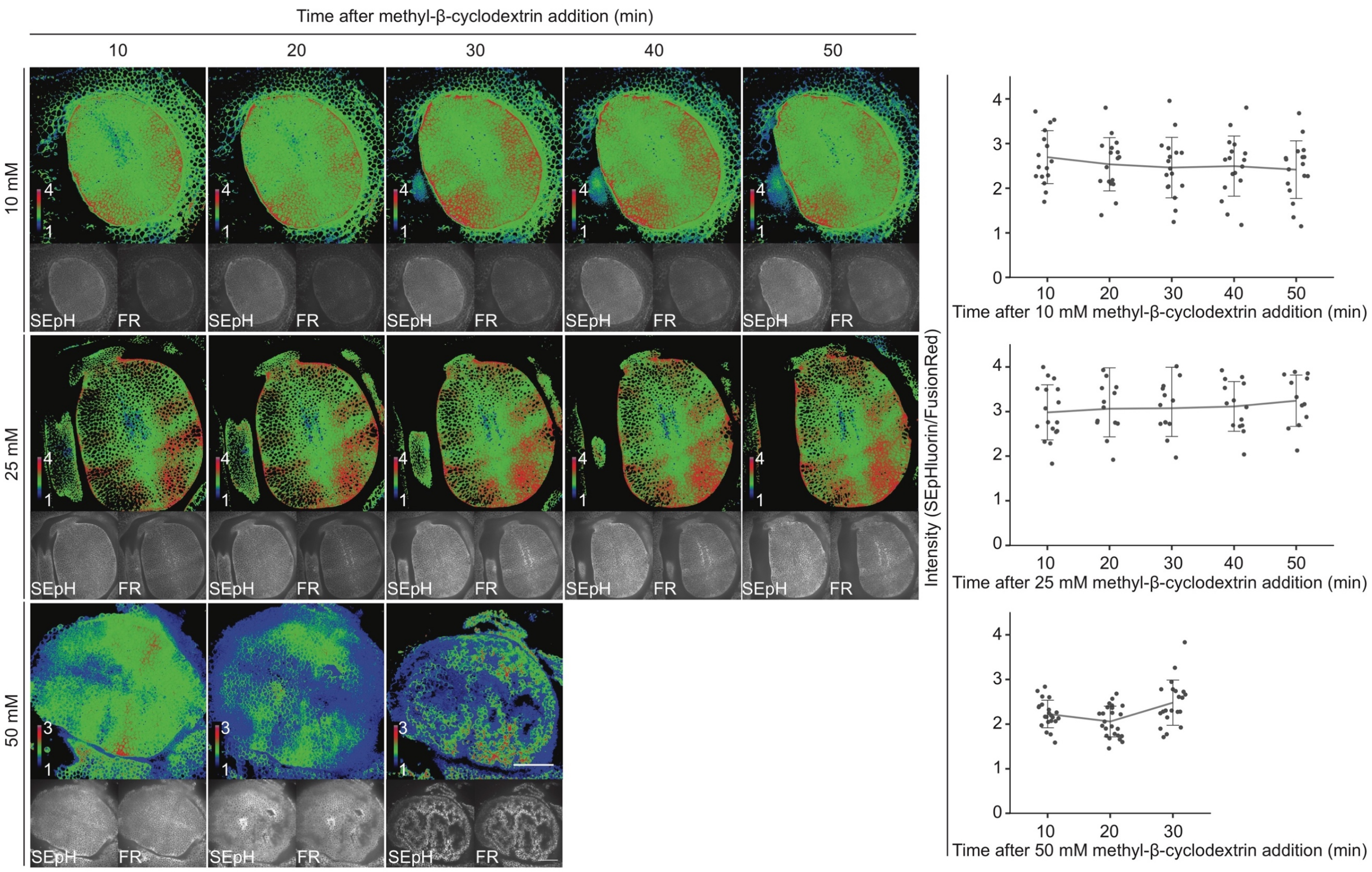

Figure R2. Effect of MβCD on tpHusion membrane localization. SEpHluorin (SEpH), FusionRed (FR) and ratiometric images of wing pouches incubated in indicated concentrations of MβCD for 10-50 minutes. n > 7 larvae. Data are represented as mean ± standard deviation. Scale bar = 50 µm.

Reference

Dason, J.S., and Charlton, M.P. (2014). A novel extraction protocol to probe the role of cholesterol in synaptic vesicle recycling. Methods Mol. Biol. 1174: 361-373.
